# Supplementary material for: Risk of Malnutrition upon Admission and after Discharge in Acutely Admitted Older Medical Patients: A Prospective Observational Study
Source: Nutrients. 2021 Aug 11;13(8):2757. doi: 10.3390/nu13082757 (PMC8398199; doi:10.3390/nu13082757)
Supplement: Supplementary file 1 [file nutrients-13-02757-s001.zip › nutrients-1305208-supplementary.pdf]

Supplementary materials

Table S1. Baseline characteristics of participants who completed follow-up and those who were lost to follow-up.

	Participants who completed follow-up		Participants who were lost to follow-up		
**Variable**	n		n		*p*-value
Age, years, median (IQR)	93	76.8 (72.2;83.0)	34	81.5 (73.4;87.0)	0.22*
Sex, male, n (%)	93	41 (44)	34	15 (44)	1
Smoking, n (%)	93	14 (15)	34	5 (15)	0.96
**Education**					0.55
1-14 years of school, n (%)	91	31 (34)	34	15 (44)	
Skilled worker, n (%)	91	45 (50)	34	15 (44)	
High School+, n (%)	91	15 (17)	34	4 (12)	
Living alone, n (%)	93	61 (66)	34	23 (68)	0.83
Body weight (kg), mean (SD)	93	75.1 (19.8)	34	77.9 (18.5)	0.48
Height (cm), mean (SD)	93	169.7 (9.3)	34	169.5 (11.7)	0.91
Body Mass Index (kg/m^2), mean (SD)	93	25.9 (5.7)	34	27 (5.1)	0.33
Quality of life (EQ-5D-5L index), median (IQR)	92	0.730 (0.552;0.833)	33	0.615 (0.426;0.705)	0.0031*
Charlson Comorbidity Index, median (IQR)	93	1 (0;2)	29	1 (0;2)	0.99*
C-Reactive Protein (mg/L), median (IQR)	93	14 (3.5;57)	34	52 (10;110)	0.013*
Maximal hand grip strength (kg), mean (SD)	90	23.6 (10.5)	34	22.1 (10.0)	0.49
Functional Recovery Score, median (IQR)	87	91 (84;99)	32	85 (73;97.5)	0.041
Physically inactive, n (%)	93	35 (38)	33	18 (55)	0.09
Fallen within previous year, n (%)	93	46 (50)	34	19 (56)	0.52
**Malnutrition or risk of malnutrition according to:**					
MNA-SF, n (%)	93	60 (65)	32	25 (78)	0.16
NRS-2002, n (%)	89	48 (54)	32	23 (72)	0.077
EVS, n (%)	92	90 (97.8)	33	32 (97.0)	1**
**Risk factors for malnutrition**					
Polypharmacy, n (%)	93	63 (68)	34	23 (68)	1
Risk of dysphagia (EAT10>=3), n(%)	92	25 (27)	32	9 (27)	0.91
Risk of depression (GDS>=2), n(%)	89	19 (20)	33	8 (24)	0.73
**Activities of daily living, n(%)**					
Need help cooking	91	20 (22)	33	9 (27)	0.54
Need help eating	92	1 (0.01)	33	0 (0)	0.55
Need help groceries shopping	92	17 (18)	33	8 (24)	0.48
**Low muscle strength in lower extremities, n (%)**					
STS-test<5 or STS-test >5 or <=8 + GS<=0.6 m/s	65	29 (31)	17	14 (41)	0.0055
**Eating Symptom questionnaire, problems with:**					
Chewing, n (%)	90	14 (15)	33	7 (21)	0.46
Diarrea, n (%)	91	24 (26)	33	10 (29)	0.67
Xerostomia, n (%)	91	54 (58)	33	22 (65)	0.46
Constipation, n (%)	91	27 (29)	33	10 (29)	0.95
Nausea, n (%)	91	36 (39)	33	14 (41)	0.77
Pain in the mouth, n (%)	90	15 (16)	33	8 (24)	0.34
Vomiting	91	15 (17)	33	10 (30)	0.09
**Cognitive impairment**					
Moderately impaired (OMC= 7-10), n (%)	123	49 (54)	33	18 (54.5)	0.66
Severely impaired (OMC >10) , n (%)	123	22 (24)	33	6 (18.2)	

*Wilcoxons-Rank-Sum-Test
**Fisher's Exact Test

Table 2S. Answers to Mini Nutritional Assessment - Short form questions at baseline and follow-up

		At baseline, n (%)	At follow-up, n (%)
**Normal nutritional status**		30 (24)	21 (23)
**At risk of malnutrition**		54 (44)	41 (45)
**Malnourished**		40 (32)	29 (32)
**Question**	**Answer**	**At baseline, n (%)**	**At follow-up, n (%)**
Weight loss during the last 3 months	0 = weight loss greater than 3 kg	33 (26)	28 (30)
	1 = does not know	4 (3)	2 (2)
	2 = weight loss between 1 and 3 kg	18 (14)	15 (16)
	3 = no weight loss	70 (56)	48 (52)
Mobility	0 = bed or chair bound	6 (5)	4 (4)
	1 = able to get out of bed / chair but does not go out	30 (24)	25 (27)
	2 = goes out	89 (71)	64 (69)
Has suffered psychological stress or acute disease in the past 3 months?	0 = yes	109 (87)	85 (91)
	2 = no	17 (13)	8 (9)
Neuropsychological problems	0 = severe dementia or depression	4 (3)	4 (4)
	1 = mild depression	0 (0)	0 (0)
	2 = no psychological problems	122 (97)	88 (96)
Body Mass Index (BMI)	0 = BMI less than 19	10 (8)	10 (11)
	1 = BMI 19 to less than 21	12 (10)	7 (8)
	2 = BMI 21 to less than 23	15 (12)	19 (20)
	3 = BMI 23 or greater	90 (71)	57 (61)

Table 3S, Answers to the Nutritional risk screening 2002 questions at baseline and follow-up

**Primary screening**	**Points - description**	**Baseline, n(%)**	**Follow-up, n(%)**
BMI>20.5?	1=Yes	19 (15)	15 (16)
	0=No	107 (85)	78 (84)
Weight loss within the last three months?	1=Yes	52 (41)	44 (48)
	0=No	74 (59)	48 (52)
Decreased dietary intake within the last week?	1=Yes	72 (58)	26 (28)
	0=No	53 (42)	67 (72)
Critically ill?	1=Yes	2 (2)	0 (0)
	0=No	121 (98)	85 (100)
Primary screening score	0	39 (32)	35 (42)
	1	37 (30)	26 (31)
	2	35 (29)	18 (21)
	3	12 (10)	5 (6)
	4	0 (0)	0 (0)
**Secondary screening,** performed only in those with at primary screening score>1			
Malnutrition score*	0 – No malnutrition	2 (2)	13 (24)
	1 – Mild malnutrition	34 (41)	18 (33)
	2 – Moderate malnutrition	24 (29)	13 (24)
	3 – Severe malnutrition	23 (28)	10 (19)
Severity of disease**	0 – No disease that increases nutritional requirements	9 (11)	37 (70)
	1 – Disease that induces light increment of nutritional requirements	68 (80)	13 (25)
	2 – Disease that induces moderate increment of nutritional requirements	8 (9)	3 (6)
	3 – Disease that induces large increment of nutritional requirements	0 (0)	0 (0)
Age correction	1 – Age≥70 years	74 (86)	48 (87)
	0 – Age<70 years	12 (14)	7 (13)
Total Secondary screening score	0	0 (0)	1 (2)
	1	3 (4)	11 (21)
	2	8 (10)	15 (29)
	3	29 (35)	10 (19)
	4	20 (24)	12 (23)
	5	17 (21)	3 (6)
	6	5 (6)	0 (0)
	7	0 (0)	0 (0)

* 1 is defined as a weight loss larger than 5% of body weight within the last 3 months or a dietary intake between 50% and 75% of nutritional requirements within the last week. 2 is defined as a weight loss larger than 5% of body weight within the last 2 months or a dietary intake between 25% and 50% of nutritional requirements within the last week or a BMI between 18,5 and

20,5 and reduced general health state. 3 is defined as a weight loss larger than 5% of body weight within the last 1 months or a dietary intake between 0% and 25% of nutritional requirements within the last week or a BMI below 18,5 and reduced general health state.
** Examples of each caterogy; 1: hip fracture, chronic disease with acute complications (eg. COPD), chronic dialysis, diabetes, cancer; 2: larger surgery, postoperative acute renal failure, apoplexy, Severe pneumonia; 3: Head injury, burn injury larger than 50% of body surface, severe infections (sepsis), patients in intensive therapy.

Table 4S, Answers to questions of the Eating validation Scheme at baseline and follow-up

Question	Answer<br>Points - description	Baseline, n (%)	Follow-up, n (%)
**Part 1. Eating habits**			
1. How many meals do you eat each day?	0 – 3 or more	89 (70)	75 (81)
	1 – 2 or less	37 (29)	18 (19)
2. How many slices of bread do you eat each day? (1 slice of bread id e.g., ½ slice of rye bread, 1 slice of wheat bread, ½ of a bun)	0 – 4 or more	24 (19)	18 (19)
	1 – 2 pieces or less	31 (25)	27 (29)
	* – between 2 and 4	71 (56)	48 (52)
# How many potatoes do you eat at a hot meal (rice or spaghetti in equal amounts)?	0 – more than 2	81 (64)	55 (59)
	1 – 2 or less	44 (35)	38 (41)
How many glasses of milk do you drink each day (incl. cocoa)?	0 – more than 1 glass	48 (38)	24 (26)
	* – 1 glass or less	78 (61)	69 (74)
# How often do you have cheese or fermented milk products?	0 – 2 slices/portions or more every day	70 (55)	54 (58)
	1 – less than 2 slices/portions every day	54 (43)	39 (42)
Leaves 25% or more of food uneaten at most meals	0 - No	59 (47)	62 (67)
	1 - Yes	67 (53)	31 (33)
**Involuntary weight loss within the last month**	0 - No	78 (61)	59 (63)
	1 - Yes	48 (38)	34 (37)
**Part 2. Are any of the listed nutritional risk factors present?**			
Chewing or swallowing problems?	No	85 (67)	24 (26)
	Yes	40 (32)	69 (74)
Eating dependency?	No	101 (80)	77 (84)
	Yes	25 (20)	15 (16)
Acute disease or acute change in chronic disease?	No	9 (7)	82 (89)
	Yes	117 (93)	10 (11)
**Part 1, n with 1 point or more.**	1 point	108 (86)	73 (79)
**Part 2, n with a yes in one of the questions.**	1 point	121 (96)	35 (38)
**Total (SUM of part 1 and 2)**	0 points – no risk of malnutrition	3 (2)	14 (15)
	1 point- at risk of malnutrition	16 (13)	49 (53)
	2 points – will benefit from a nutritional intervention	106 (85)	29 (32)

*Check next question marked with a #
